# *In silico*-based screen synergistic drug combinations from herb medicines: a case using *Cistanche tubulosa*

**DOI:** 10.1038/s41598-017-16571-3

**Published:** 2017-11-27

**Authors:** Jianling Liu, Jinglin Zhu, Jun Xue, Zonghui Qin, Fengxia Shen, Jingjing Liu, Xuetong Chen, Xiaogang Li, Ziyin Wu, Wei Xiao, Chunli Zheng, Yonghua Wang

**Affiliations:** 1 Key Laboratory of Resource Biology and Biotechnology in Western China, Ministry of Education, School of Life Sciences, Northwest University, Xi’an, China; 2 Lab of Systems Pharmacology, Center of Bioinformatics, College of Life Science, Northwest A&F University, Yangling, China; 3 State Key Laboratory of New-tech for Chinese Medicine Pharmaceutical Process, Jiangsu Kanion Parmaceutical Co. Ltd, Lianyungang, China

## Abstract

Neuroinflammation is characterized by the elaborated inflammatory response repertoire of central nervous system tissue. The limitations of the current treatments for neuroinflammation are well-known side effects in the clinical trials of monotherapy. Drug combination therapies are promising strategies to overcome the compensatory mechanisms and off-target effects. However, discovery of synergistic drug combinations from herb medicines is rare. Encouraged by the successfully applied cases we move on to investigate the effective drug combinations based on system pharmacology among compounds from *Cistanche tubulosa* (SCHENK) R. WIGHT. Firstly, 63 potential bioactive compounds, the related 133 direct and indirect targets are screened out by Drug-likeness evaluation combined with drug targeting process. Secondly, Compound-Target network is built to acquire the data set for predicting drug combinations. We list the top 10 drug combinations which are employed by the algorithm Probability Ensemble Approach (PEA), and Compound-Target-Pathway network is then constructed by the 12 compounds of the combinations, targets, and pathways to unearth the corresponding pharmacological actions. Finally, an integrating pathway approach is developed to elucidate the therapeutic effects of the herb in different pathological features-relevant biological processes. Overall, the method may provide a productive avenue for developing drug combination therapeutics.

## Introduction

Neurogenic neuroinflammation is defined as orchestrated actions of innate and adaptive immune cells, vascular cells and neurons triggered by pathological states and enhanced neuronal activity in the central nervous system (CNS). It is likely to play a role in priming of CNS inflammatory reactions by conditions such as pain, psychological stress, and epilepsy or become a pathogenic factor in neurodegenerative diseases. The current agent for the treatment of neuroinflammation mostly belong to monotherapy, including dopamine, somatostatin, neuropeptide, or adenosine and so on. However, for example, long-term use of the common but old drugs COX inhibitors, Nonsteroidal Anti-inflammatory Drugs (NSAIDs), might cause adverse side-effects, gastrointestinal lesions or cardiovascular risks^1,2^ and clinical trial results remain unsatisfactory particularly^3,4^, so there is an unmet need for new treatments of neuroinflammation.

The novel therapy of drug combinatorial may be a surging strategy to meet the needs of the development of the novel drugs as well as overcome hurdles in treatments of complex diseases. Combination or multicomponent therapy could fulfill the above requirements, in which two or more drugs are used together^5^, with the listed advantages: higher efficacy, minimal cross-resistance, low dose while fewer side effects, and less toxicity when compared to single-drug agent^6^. It has been used in the treatment of complex diseases^7,8^ for nearly 30 years. Intriguing, drug combination therapy is also applied in the researches on neurological diseases, for instance, combination of glimepiride and ibuprofen could effectively reduce inflammation in Alzheimer Disease (AD)^9^ or ketamine/atropine might lower pro-inflammatory proteins expression in epileptic mice^10^. Synergistic drug combinations may therefore bring new inspiration for tracing effective treatments for neuroinflammation. Hence, how can we crack the hard nut to achieve the optimal combinatorial drugs?

Nowadays, the existing approaches to screen out drug combinations are as follows, systematic surveys of drug pairs *in vitro* such as the high throughput screening method^11^ and the ‘*M*ultiplex *S*creening for *I*nteracting *C*ompounds’ (*MuSIC*)^12^ or evaluations of the pairwise drug combinations with large-scale experiments^13,14^. Nevertheless, the excessive consumption of manpower, natural resources and time of arduous empirical testing may be an inevitable issue in the evaluation of effective drug combinations. In response to conquering the shortcomings, approaches based on network analysis, especially genetic interaction networks^15^, chemical systems biology data^16^, and molecular and pharmacological data^17^ are also emerged for combinatorial drug discovery. Furthermore, an increasing body of investigators^18,19^ proposed novel network approaches to predict optimal combinations and offered the corresponding experimental validation in the meantime, so integrating network prediction with experimental validation may be a new trend in the field of combinational prediction.

Herbal medicines involve considerable numbers of formulas (*Fang-Ji* in Mandarin) and chemical ingredients, which forms a natural products database, so that it could afford innovative clues, fundamental biology data for the development of drug combinations, as Professor Li^20,21^ and Professor Liu^22^ introduced holistic analysis methods based on integrated biology to decipher the molecular mechanisms of herbal medicines: Liu-Wei-Di-Huang pill or Reduning Injection. In our previous work, we found not only two representative herbs *Lonicera japonica* and *Fructus Forsythiae* show synergistic effects on influenza or inflammation^23^, but also compounds rutin and amentoflavone present synergistic effects in preventing depression^24^.

In our current work, we develop a system pharmacology approach to discover the synergistic drug combinations among compounds from the herb *Cistanche tubulosa* (SCHENK) R. WIGHT, the following steps are proposed: firstly, we pick out bioactive compounds through drug-likeness prediction, which are used as baits to fish the related targets. And then, inspired by “network target”-based paradigm to prioritize synergistic agent combinations in a high throughput way^25^, we acquire effective drug combinations among the potential compounds based on an in-house algorithm that is termed Probability Ensemble Approach (PEA)^26^ with high training efficiency, extensive applicability and two quantitative indexes to describe the property of a drug combination. Finally, we use the obtained targets and the compounds of candidate pairs to build network/pathway and then provide analysis to encode the mechanism of *Cistanche tubulosa* on neuroinflammation holistically. As an example, it is the first time to screen out effective drug combinations from natural products based on system pharmacology through integrating computational methods and experimental validation to approve the reliability of the prediction. We believe that this may help to personalize neuroinflammation treatment, enhance our understanding of effective neuroprotective development and will aid future preclinical research.

## Results

### Targets of *Cistanche tubulosa*

To get the targets related to neuroinflammation we firstly achieve the ingredients in *Cistanche tubulosa* (SCHENK) R. WIGHT by searching the TCMSP database (http://lsp.nwu.edu.cn/), which results in 103 compounds (Supplementary Table S1, See Materials and Methods). Then, we analyze their drug-likeness by applying the DL prediction model constructed in our previous work (See Materials and Methods). In this way, we achieve 63 potential bioactive compounds (Supplementary Table S2) with DL index ≥0.18. Subsequently, by means of the SysDT and WES algorithms, we identify 117 targets of these potential bioactive compounds. Finally, 43 potential targets (Supplementary Table S3) closely related to neuroinflammation are retrieved after deleting noise and errors, through mapping the 117 targets of the compounds to the CTD database.

### GOBP enrichment analysis for targets

**Figure 1:**
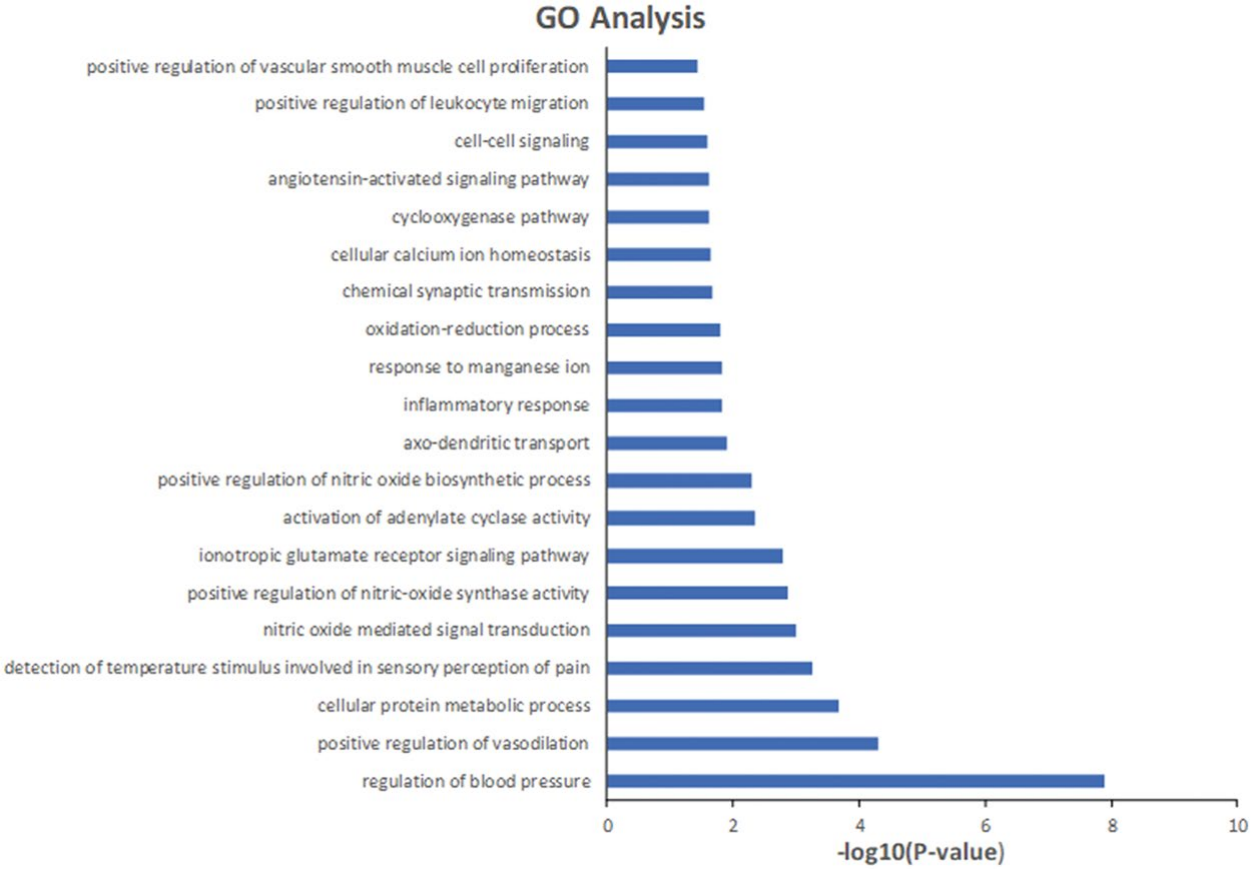
Gene Ontology (GO) analysis of potential target genes. The y-axis shows significantly enriched ‘Biological Process’ (BP) categories in GO of the target genes, and the x-axis shows the enrichment scores of the terms (*P*-value ≤ 0.05).

To check whether the proteins targeted by the potential bioactive compounds are closely related to neuroinflammation, we perform GOBP enrichment analysis through mapping targets to DAVID. Fig. 1 shows a GO tree representing the results of significantly enriched GOBP terms (*P* value ≤ 0.05), where the targets are categorized into 20 different groups, such as positive regulation of vascular smooth muscle cell proliferation and positive regulation of leukocyte migration. Among these groups, chemical synaptic transmission, inflammatory response, cell-cell signaling and so on are all closely associated with neuroinflammation. For instance, the synaptic alterations occurring during neuroinflammatory diseases are largely mediated by inflammatory cytokines released from infiltrating T cells and from activated microglia, and are responsible, at least in part, for irreversible dendritic pathology. Collectively, these observations suggest that the predicted targets probably can contribute to the treatment of neuroinflammation.

### Compound-Target network construction and analysis

**Figure 2:**
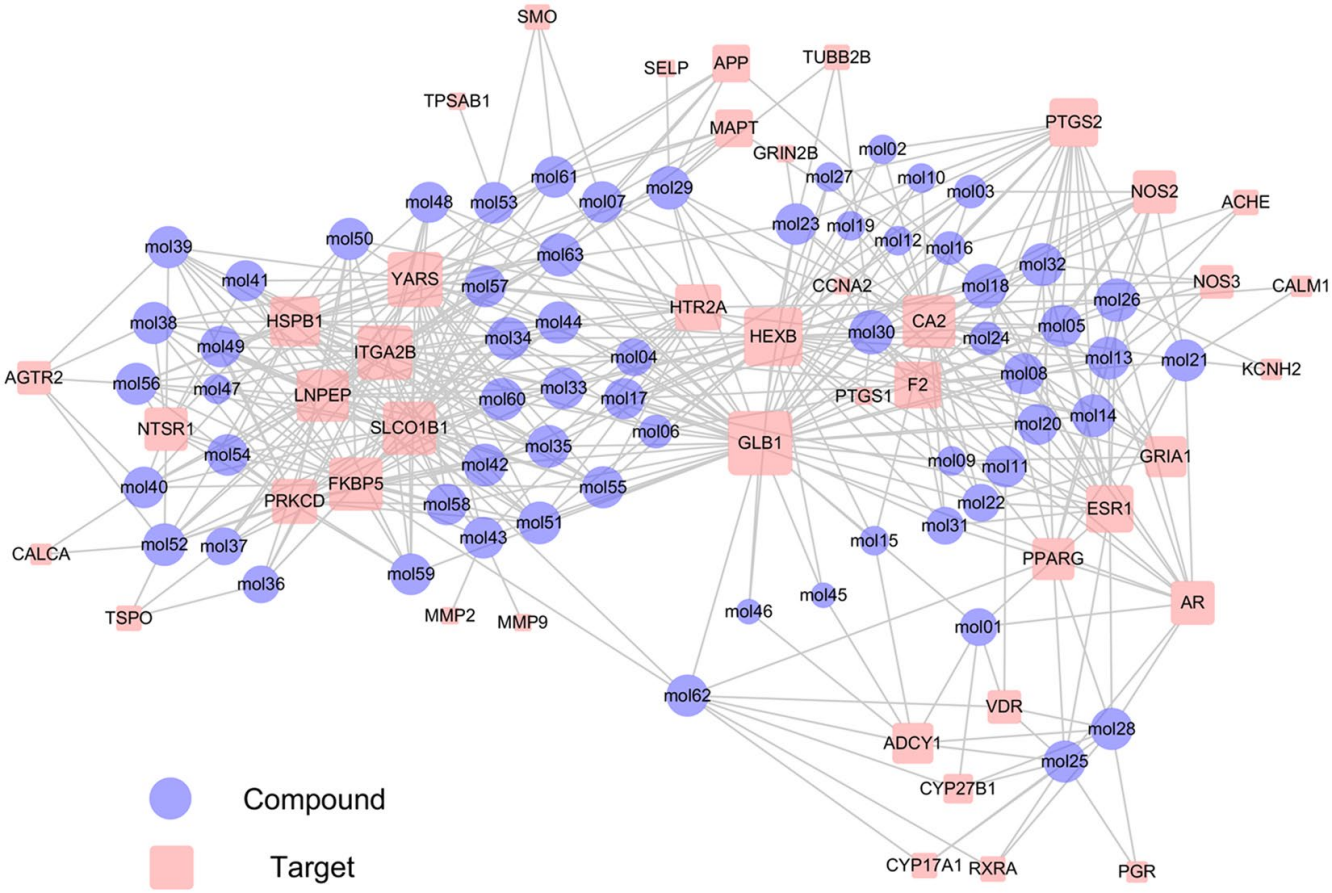
C-T network. A compound and a target node are linked if the protein is targeted by the corresponding compound. Node size is proportional to its degree and the letters are node labels.

Generally, the feasible and effective combination therapies are combinations of bioactive compounds with ideal pharmacokinetic properties, which can balance the disease network by regulating specific targets. Therefore, we further construct a static compound-target network to check their topological relations. As shown in Fig. 2, the bipartite compound-target (C-T) network exhibits 482 interactions between 63 compounds and 43 targets in a visually appealing manner. We analyze the nodes’ degree in the compound-target network which resulting in an average degree per compound of 11.209 and 7.651 per target, respectively. We observe that among the 63 compounds, 38 of them adjust more than 7 targets (larger than the average degree), manifesting the potential synergistic effects among them.

For example, syringin (mol30) interacts with the largest number of targets, which may play a hub role in the network. Interestingly, a study proves that the phenolic constituent syringin isolated from *Euonymus alatus* (Thunb.) Sieb. (Celastraceae) has an anti-neuroinflammatory effect by inhibiting NO production^27^. 2′-acetylacteoside (mol42) is in control of 10 different targets (degree = 10). Among these targets, HSPB1 (also known as HSP27), as an example, is a molecular chaperone that displays neuroprotective properties in many disease and injury models^28,29^. For verbascoside (mol33), the neuroprotective properties of this bioactive compound involve modulation of transcription factors and consequent altered gene expression, resulting in downregulation of inflammation^30^. It is worth noting that although the topology property of the network does not bias toward echinacoside (mol41, degree = 7), it is a potential novel orally active compound for regulating neuroinflammation and related signals in Parkinson’s disease and may provide a new prospect for clinical treatment. Taken together, these results indicate that the screened potential active compounds are all related to neuroinflammation and can be regarded as the data set for predicting drug combinations.

### Compound-Target-Pathway network construction and analysis

**Figure 3:**
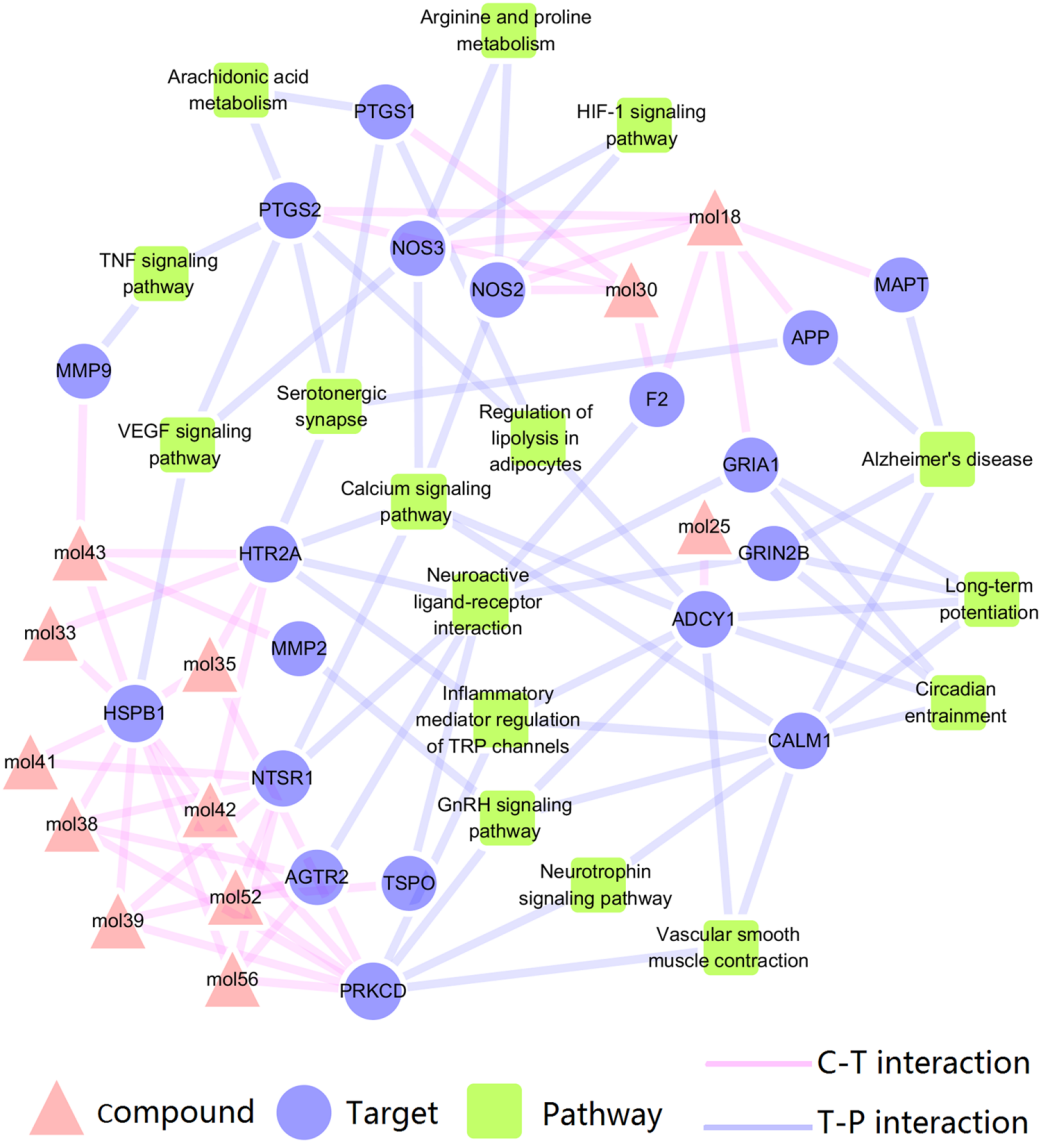
C-T-P network. The link is placed between a target and a compound of the 10 different drug combinations if the compound is lighted at the target. The link is placed between a target and a pathway if the pathway is lighted at the target. The information of pathways is obtained by mapping the target proteins to the KEGG pathway database. The letters are node labels.

By employing the PEA algorithm, we get 10 different drug combinations (Table 1.), which involve in 12 compounds. These drug combinations are all with high synergy probability, which represents the possibility of inducing synergy between two compounds. Therefore, up to a certain extent, these compounds are considered to be the pharmacological fundamental substances of *Cistanche tubulosa*. Based on the target information of these compounds and the pathways derived from DAVID, we construct the compound-target-pathway (C-T-P) network to preliminarily light on the molecular mechanisms of these compounds. The resulted compound-target-pathway network consists of 47 nodes and 99 edges. As illustrated in Fig. 3, these compounds can act on not only the proteins of the upstream but also the downstream of the pathways associated with neuroinflammation, especially the direct indicators of inflammation markers.

**Table 1:** Synergy probabilities of the top 10 pairs.

Compound 1	Compound 2	Synergy probability
echinacoside	verbascoside	0.97
isoacteoside	2′-acetylacteoside	0.95
echinacoside	2′-acetylacteoside	0.92
cistansinenside A	tubuloside A	0.86
cistantubuloside B1	cistantubuloside A	0.85
β-sitosterol	2′-acetylacteoside	0.82
kankanoside O	kankanoside K1	0.79
tubuloside A	syringin	0.73
cistansinenside A	kankanoside K1	0.67
kankanoside O	verbascoside	0.61

In particular, the vast majority of compounds target the upstream proteins such as HSPB1, HTR2A, NTSR1 and others, indicating the macro regulation of these compounds for the treatment of neuroinflammation. For instance, compound echinacoside (mol41) targets the VEGF pathway through protein HSPB1(Supplementary Figure 1). Fortunately, studies show that HSPB1 has significant cytoprotective properties in several models of neurological disease *in vivo* ^31,32^ or *in vitro* ^33^ and it may play a role in anti-inflammatory effect by regulating the nuclear factor-κB (NF-κB) signaling pathway^34,35^. In addition, a study also demonstrate that R-Ras could regulate angiogenic activities of endothelial cells in part via inhibition of the p38 mitogen-activated protein kinase (p38 MAPK)-HSPB1 axis of the VEGF signaling pathway^36^.

We can get that compound tubuloside A (mol56) target the Calcium signaling pathway through protein NTSR1 in the network. It is all known that calcium ions (Ca^2+^) is a universal second messenger in the immune system cells. The reduction in synaptic activity or increased extrasynaptic N-methyl-D-aspartic acid (NMDA) receptor signaling may lead to nuclear calcium dyshomeostasis^37,38^, thereby increasing the occurance of neurodegeneration and cognitive dysfunction. Furthermore, the target protein NTSR1 is a member of the large superfamily of G-protein coupled receptors, and signaling is generated through binding G proteins that may activate a phosphatidylinositol-calcium second messenger system and downstream MAP kinases^39^.

Another example is that compound 2′-acetylacteoside (mol42) target the GnRH signaling pathway through protein PRKCD. PRKCD, one of the PKC isoforms, is a major mediator of the activation of extracellular regulated protein kinases1/2 MAPK (ERK1/2 MAPK), c-Jun N-terminal protein kinases MAPK (JNK MAPK) and p38 MAPK by gonadotropin releasing hormone (GnRH)^40^. And, as key processes in the GnRH-stimulated signaling network, the downstream MAPK cascades and arachidonic acid(AA) metabolites^41^ could produce inflammatory protein, COX-2. As for kankanoside O (mol18) and syringin (mol30), they interact directly with the downstream proteins PTGS2, NOS2 and other inflammatory indicators, which is an intuitive reflect of the compounds’ efficacy. A study shows syringin could suppress the production of tumour necrosis factor-α (TNF-α) in Lipopolysaccharides (LPS)-stimulated RAW264.7 cells^42^. And the other studies demonstrate that syringin could also lower NO concentration and NOS activity^43^ or the production of prostaglandin E2^44^. Isoacteoside (mol43) targeted MMP9, is an important player in central nervous system and would be a putative mediating enzyme for neuropsychiatric disorders such as schizophrenia and bipolar illness^45^. The degradation of NF-κB and phosphorylation of p38, ERK1/2, JNK MAPK or Akt (Protein Kinase B) of the upstream signaling pathways could modulate the MMP9 gene expression and inhibition of MMP9 that may reduce the expression of inducible nitric oxide synthase (iNOS) in activated cells^46–48^.

### Pathway analysis

**Figure 4:**
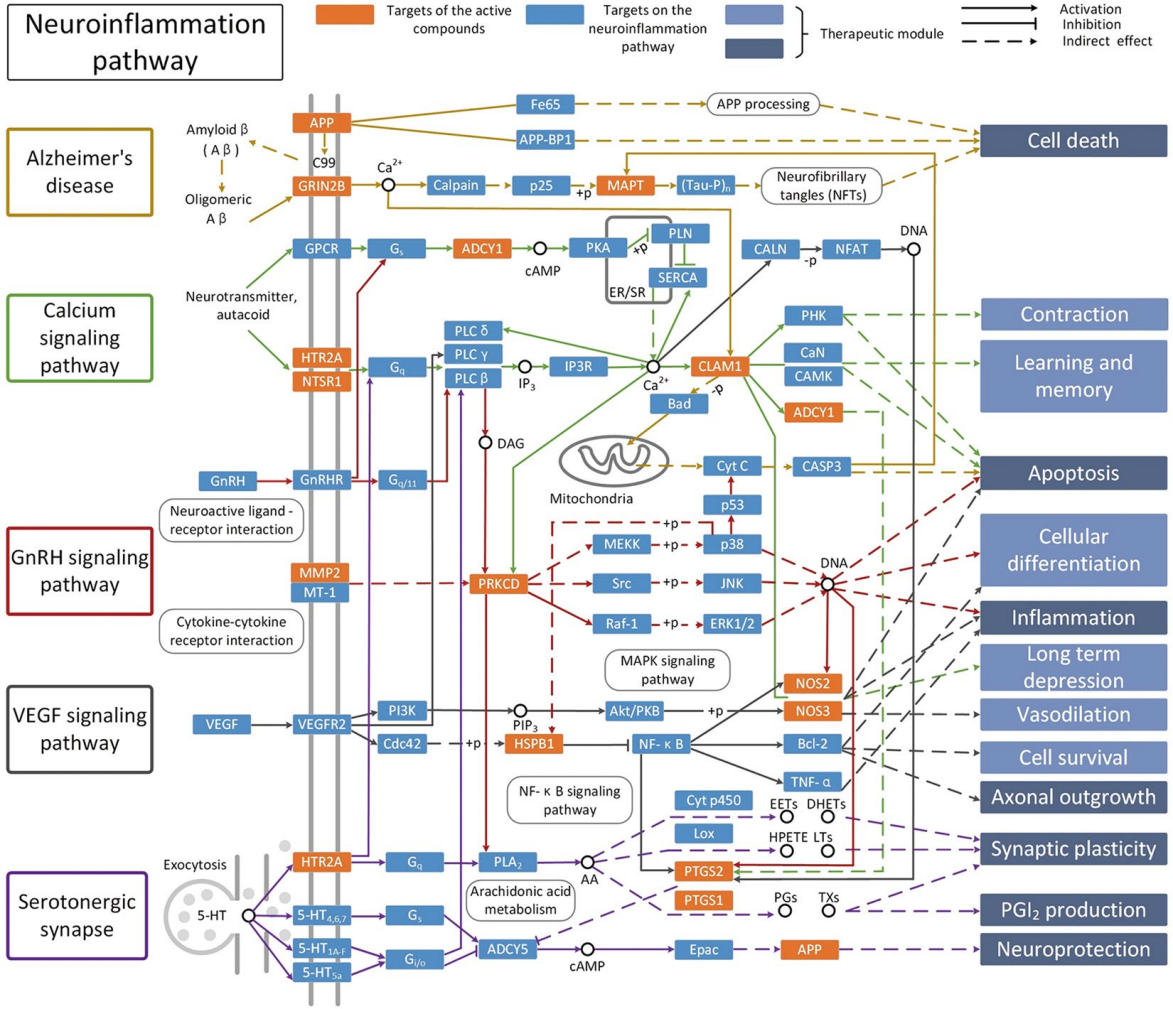
Neuroinflammation pathway and therapeutic modules.

An incorporated “Neuroinflammation pathway” is constructed by integrating the key pathways that obtained through compound-target-pathway network analysis, including the Alzheimer’s disease pathway, Calcium signaling pathway, GnRH signaling pathway, VEGF signaling pathway and the Serotonergic synapse. Of the 43 targets, 19 can be mapped onto the “Neuroinflammation pathway”. As shown in Fig. 4, the *Cistanche tubulosa* represents the targets of the active compounds that distribute in the “Neuroinflammation pathway”. The “Neuroinflammation pathway” is classified into 13 different therapeutic modules, such as cell death, apoptosis, inflammation and neuroprotection. In this study, we take cell death, inflammation and neuroprotection modules as examples to clarify the mechanism of *Cistanche tubulosa* for neuroinflammation.

#### Cell death module

Targets locate in the Alzheimer’s disease pathway mainly involve in cell death process, suggesting that cell death is closely related to neuroinflammation. Studies demonstrate that neuroinflammation mediated by microglia contributes to neuronal cell death, which is not restricted to a specific disease but implicated in various diseases such as ischemia^49^, Parkinson’s disease^50^ and Alzheimer’s disease^51^. As shown in Fig. 4, targets APP, GRIN2B and MAPT are regulated by the active compounds from C*istanche tubulosa* indicating that they can inhibit cell death and thereby offer a treatment for neuroinflammation. Interestingly, APP intracellular domain impairs adult neurogenesis in transgenic mice by inducing neuroinflammation^52^. Inability of MAPT properly regulate neuronal microtubule dynamics and thus mediate neuronal cell death^53^. All these indicate that *Cistanche tubulosa* may treat neuroinflammation through inhibiting cell death.

#### Inflammation module

The term neuroinflammation is the inflammatory reactions in the CNS in response to neuronal activity^1^. In this study, we detect that GnRH signaling pathway and the VEGF signaling pathway involve in the inflammatory module (Fig. 4). For instance, MMP2 in the GnRH signaling pathway belongs to the MMPs family, which are expressed in physiological situations and pathological conditions involving inflammation. And MMPs regulate several functions related to inflammation including bioavailability and activity of inflammatory cytokines and chemokines^54^. In addition, the VEGF signaling pathway can lead to the generation of NOS2, moreover, it participates in the acute inflammatory response to LPS by multiple mechanisms: involvement in proinflammatory cytokine signaling and alteration of the expression of various genes that affect inflammatory-immune responses to LPS^55^. Collectively, all these indicate that *Cistanche tubulosa* may cure neuroinflammation by regulating the inflammatory system.

#### Neuroprotection module

Neuroprotection is the mechanisms and strategies used to protect against neuronal injury or degeneration in the CNS following acute disorders (e.g. stroke or nervous system injury/trauma) or as a result of chronic neurodegenerative diseases (e.g. Parkinson’s, Alzheimer’s, Multiple Sclerosis). The goal of neuroprotection is to limit neuronal dysfunction/death after CNS injury and attempt to maintain the highest possible integrity of cellular interactions in the brain resulting in an undisturbed neural function. As can be seen from the Fig. 4, some targets on Serotonergic synapse pathway involves in the function of neuroprotection. For example, the production of prostaglandins through PTGS1 and PTGS2 (also known as COX-1 and COX-2) is an essential mediator in evoking anti-inflammatory and novel pro-resolving mechanisms^56^. A recent study has shown that gene expression of ADCY5, an enzyme which catalyzes the generation of cAMP^57^, is reduced by promoter methylation in COX-2-induced human HCC cell lines^58^. Based on the above analysis, we speculate that COX-2 accumulation may influence the secretion of sAPPα, the α cleavage of APP cleaved by α-secretase that is modulated by cAMP and further exert the neuroprotective effect^59,60^. Our results show that neuroprotection plays an important role in the treatment of neuroinflammation.

## Experimental validation

### The viability of BV2 microglia cells treated by the compounds

**Figure 5:**
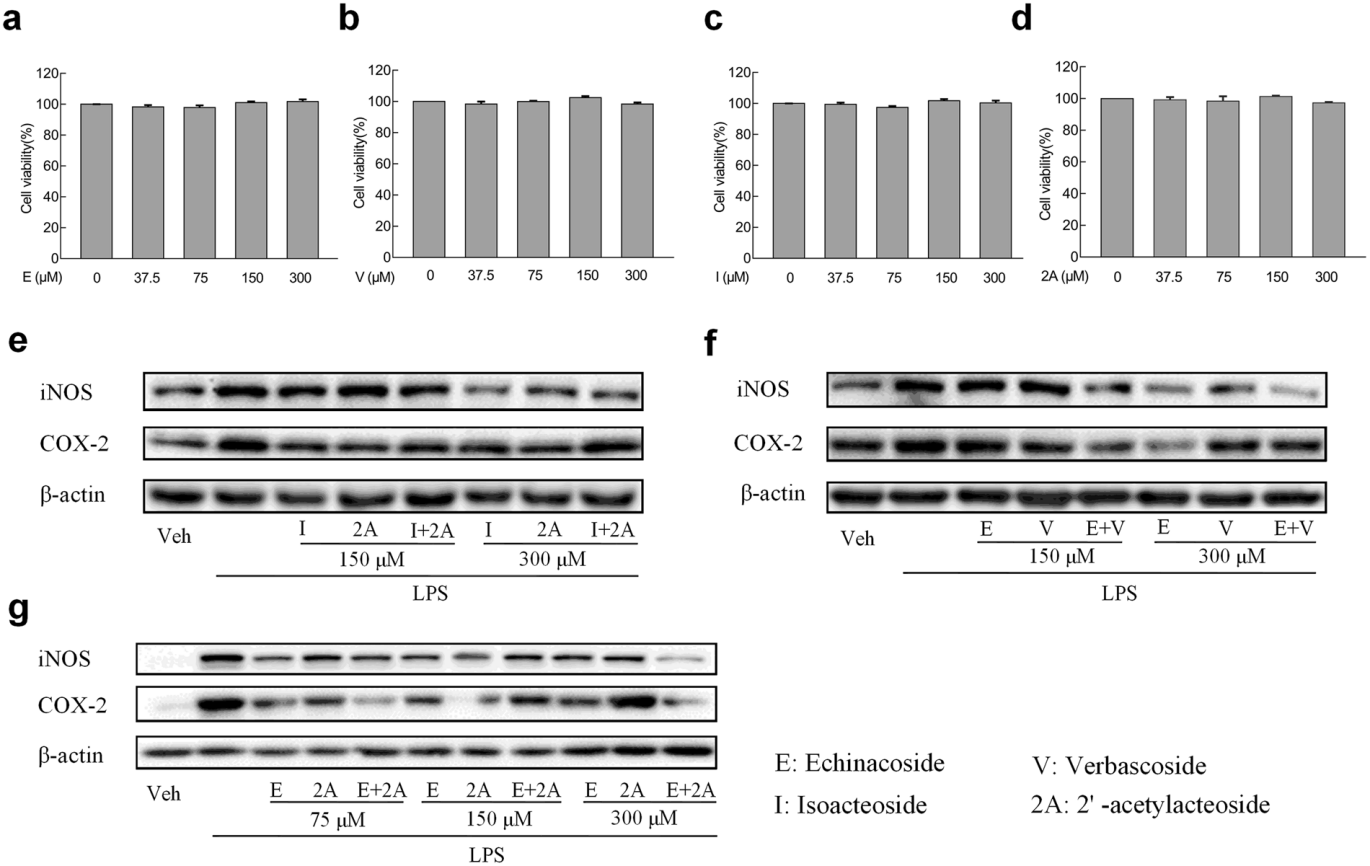
Cell viability and inhibition of iNOS and COX-2 in BV2 cells. (**a**–**d**) Cell viability of BV2 cells. The determination of cell viability of BV2 cells is carried out by CCK-8 assay after treated with control or (**a**) Echinacoside (E), (**b**) Verbascoside (V), (**c**) Isoacteoside (I), or (**d**) 2′-acetylacteoside (2A) (37.5, 75, 150, or 300 μM) for 24 h. No significant differences are found among groups. (**e**–**g**) BV2 cells are pretreated with (**e**) I or 2A (150 or 300 μM), (**f**) E or V (150 or 300 μM), and (**g**) E or 2A (75, 150 or 300 μM) or the combinations for 2 h, vehical as the control. Then exposure to LPS (1 μg/ml) for 18 h, then iNOS and COX-2 accumulation of cytoplasm are measured by western blot. β-actin is used as loading control. All results are repeated at least three independent experiments with the same tendency.

BV2 microglia cells (8 × 10^4^ cells/ml) are fed with the concentrations of 37.5 to 300 μM per milliliter culture media with no serum of the four compounds for 24 h. We regard the cell viability of control group cultured in the absence of serum with less than 0.1% DMSO as 100% (Fig. 5(a–d)). Apparently, no significant cellular cytotoxicity is observed at the prescribed dosages of the groups.

### Validation of drug synergy and potential anti-inflammatory effect ***in vitro***

To further assess the obtained results *in silico*, four compounds covering three synergistic pairs, namely isoacteoside, 2′-acetylacteoside, echinacoside and verbascoside, are selected to examine their drug synergistic effects and potential anti-inflammatory effect using BV2 cells treated with LPS. In particular, we conduct western blot analysis for iNOS and COX-2 protein expression to conform the synergy and anti-inflammatory effects of the predicted drug combinations.

As shown in Fig. 5(e–g), the levels of iNOS and COX-2 proteins in the panel of BV2 cell lines tested are reported. We observe that either isoacteoside or 2′-acetylacteoside treatment, the protein expressions of iNOS and COX-2 in BV2 cells are both declined significantly at different dose levels. However, treatment with the combination of isoacteoside and 2′-acetylacteoside induce a significant increase of the inflammatory factors iNOS and COX-2 (Fig. 5(e)). Figure 5(f) illustrates that echinacoside or verbascoside treatment, as a single agent, causing a decrease of the iNOS and COX-2 expression. Moreover, as expected, treatment with echinacoside in combination with verbascoside at the concentration of 150 μM resulted in a more pronounced decrease in the levels of protein expression (iNOS and COX-2), indicating the synergistic anti-inflammatory effects of this drug combination. Similarly, as indicated in Fig. 5(g), the combination of echinacoside and 2′-acetylacteoside show a significant synergistic effect on the inhibition of COX-2 at the concentration of 75 μM or 300 μM. However, for iNOS, at the dosage of 300 μM, the combination represents marked suppression of the protein. In contrast, we find that there are no obvious inhibition effects on both iNOS and COX-2 about the combination of isoacteoside and 2′-acetylacteoside or echinacoside and verbascoside at the concentration of 75 μM (Supplementary Figure 2), besides, treatment with other pairs shows a much weaker effect compared with the single agents at the dosage of 150 or 300 μM that can be seen in Fig. 5 (e–g).

To sum up, the *in vitro* study provides additional information for screening drug combinations with potentially anti-inflammatory effect and demonstrates the reliability of *in silico* screen strategy.

## Discussion

Neuroinflammation is implicated in the majority of neurological, psychiatric and neurodevelopmental diseases due to that is not only a consequence but could be a trigger of the pathology^61^. However, current treatments for neuroinflammatory are monotherapies mostly, limited by well-known side effects as we know, COX-2 inhibitors may lead cardiovascular defects responded to long-term treatment, and TNF-targeted treatment could cause infection through immunosuppression^62^. Combinatorial therapeutic approaches may be imperative to improve treatment of complex diseases with the following advantages: the countered network robustness and bypass compensation, the increased clinical efficacy while maintaining minimal human toxicity and the reduced dosage of each compound^63^. However, exploration the synergistic drug combinations among compounds derived from herb medicines based on system pharmacology is few restricted by the possible main reason of large amounts of compounds.

In the work, we firstly gain 63 potential bioactive compounds from the herb *Cistanche tubulosa*, fulfilled the criteria (DL ≥ 0.18) for further analysis with the aid of the prediction which is indispensable to screen out more promising molecules with desirable property. After mapping the 133 targets of the 63 potential bioactive compounds to database, we get 43 targets related with neuroinflammation, and then GOBP clustering analysis of the predicted targets can probably contribute to the treatment of neuroinflammation. The analytical result of the C-T network displayed in an average degree per compound of 11.209 and 7.651 per target, respectively and 38 of them adjust more than 7 targets (larger than the average degree). For example, echinacoside (mol41)^64–66^ predicted with 7 targets, verbascoside (mol33)^30,67^ with 9 targets, or tubuloside B (mol57)^68,69^ with 8 targets could play key roles in neuroprotection in line with the increasing literatures.

We achieve direct therapeutic targets such as APP, MAPT (also known as Tau), PPARG^70^, MMP9, MMP2^71,72^, and HTR2A (also known as 5-HT2A), GRIN2B (glutamate ionotropic receptor NMDA type subunit 2B), and GRIA1 (glutamate ionotropic receptor AMPA type subunit 1)^73^ or downstream potential targets such as PTGS2^74^ or NOS2^75^ that are associated with neuroinflammation or various diseases of nervous system.

The analysis of the Compound-Target-Pathway network displays 12 compounds from the top 10 drug pairs through PEA algorithm, connected with the 43 potential targets and the pathways linked with neuroinflammation, for example, Calcium signaling pathway, Neuroactive ligand-receptor interaction or TNF signaling pathway and so on. In the system, these predicted compounds could act on not only the proteins of the upstream but also downstream of pathways associated with neuroinflammation and inflammatory biomarkers, in particular. Moreover, the additional information for screening drug combinations with potentially anti-inflammatory effect is provided and the reliability of *in silico* screen strategy is verified by experimental validation. Neuroinflammation pathway is comprised of the Alzheimer’s disease pathway, Calcium signaling pathway, GnRH signaling pathway, VEGF signaling pathway and the Serotonergic synapse. The analytical results distinctly explained to us that cell death, inflammation and neuroprotection modules are exemplified to decipher the mechanism of *Cistanche tubulosa* for the treatment of neuroinflammation.

Neuroinflammation accompanies various neurodegenerative diseases which could be not only a consequence but a trigger of pathology, thus, anti-inflammatory therapies are suggested to be a promising treatment approach^61^. To our disappointed, though we have realized the limitation of the monotherapies, the evaluation and the underlying mechanisms of combination therapies are still the major challenges in the development of the novel alternative strategy. This work therefore could offer new therapeutic opportunities for neuroinflammation and may open up a new avenue for discovering drug combination from natural products.

## Materials and Methods

### Compounds collection

A total of 66 chemical ingredients of *Cistanche tubulosa* are manually gathered from TCMSP (http://lsp.nwu.edu.cn/)^76^, including 26 phenylethanoid glycosides, 22 iridoids, 4 lignans, 7 monoterpene glycosides, 2 nitrogenous substances, 3 benzene acryloyl sugars, 1 sterol, 1 ketol. Given that glycosides in Cistanche tubulosa are usually hydrolyzed to liberate aglycone which is then absorbed at the intestinal mucos, thus, we take the molecules without glycoligand into consideration, which are tagged as _qt. This lead to the generation of the 103 compounds. These molecules are provided in Supplementary Table S1.

### Drug-likeness evaluation

To obtain the potential bioactive compounds from *Cistanche tubulosa*, we evaluate the drug-likeness of these ingredients by calculating the Tanimoto similarity^77^ between herbal compounds and the average molecular properties of all chemicals in the Drugbank database^78^. And, the DL prediction model has been applied successfully in many studies^79,80^ to select out bioactive compounds. In the work, DL index ≥ 0.18 of the candidates is defined as the threshold value to better suit subsequent analysis.

### Drug target prediction

The identification of the efficacy targets for leading compounds remains a key step to progress compounds into drug development^81^. Here, two in-house tools: SysDT and WES are carried out to derive the molecular target information for drug fishing. SysDT is an *in silio* model which is performed with the combination of the chemical, genomic and pharmacological information based on the two powerful mathematical tools: Random Forest (RF) and Support Vector Machine (SVM) to tackle the issue of target identification effectively^82^. The obtained model is served as a valuable platform for prediction of drug-target interactions with an overall accuracy of 97.3%, an activated prediction accuracy of 87.7% and an inhibited prediction accuracy of 99.8%. In order to capture more promising components, the filtering criteria is defined as RF value ≥ 0.7 or SVM ≥ 0.8 in this study.

Weighted ensemble similarity (WES) is a new powerful computational model to pinpoint the drug direct targets of the actual bioactive ingredients^83^. As a novel tool, the obtained model performs well in predicting the binding with average sensitivity of 85% (SEN) and the non-binding patterns with 71% (SPE) with the average areas under the receiver operating curves (ROC, AUC) of 85.2% and an average concordance of 77.5%. The obtained targets are further mapped to Uniprot (http://www.uniprot.org) to normalize their names and organisms subsequently. Here, we only choose the targets of Homo sapiens for further analysis. Candidate targets of the selected compounds are mapped to the CTD database (http://ctdbase.org/)^84^ to get their related diseases and we screen out potential targets related to neuroinflammation finally.

### GO enrichment and analysis for targets

To probe the involved biological processes of the obtained targets, we map the targets to DAVID (http://david.abcc.ncifcrf.gov)^85^ and the terms with *P*-value less than 0.05 are chose in this section.

### Drug combination analysis

In our previous work, a system pharmacology framework was exploited to predict drug combinations on a newly designed model, termed Probability Ensemble Approach (PEA) with the purpose of analyzing the clinical efficacy and adverse effects of drug combinations. In detail, a Bayesian network integrating with a similarity algorithm was developed to model the combinations from compound molecular and pharmacological effect. The combined evaluation that covered the clinical efficacy and adverse effects for the predicted pairs was presented then^26^. Briefly, it shows that PEA could predict the efficacy of the pairs with high specificity and sensitivity (AUC = 0.90) in our work. In this work, we select the top ten drug combinations based on their synergy probabilities, which represents the possibility of inducing synergy between two compounds.

### Network/Pathway building and analysis

To investigate relationships between the active ingredients and the inflammatory diseases, compound-target (C-T) network and compound-target-pathway network (C-T-P) are generated by Cytoscape 2.8.1, a popular bioinformatics package for biological network visualization and data integration^86^. The quantitative properties of the network are analyzed by the two following plugins Network Analyzer and CentiScaPe 1.2. In the graphic network, nodes indicate either compounds, targets or pathways while edges encode the drug-target interaction. To further explore the biological effects of how cellular target work through modulating multiple metabolism pathways, an incorporated “pathway” is assembled in accordance with the up-to-date information of neuroinflammation pathology. Firstly, by means of mapping them onto KEGG database (http://www.genome.jp/kegg/), the achieved target profiles are aggregated into several pathways. After abandoning the indirectly sections, then, a relatively synthesized pathway is manually integrated on account of the pathological and clinical data.

## Experimental validation

### Samples preparation

Echinacoside, verbascoside, isoacteoside and 2′-acetylacteoside are purchased from Nanjing Zelang Biological Technology Co., Ltd. (Nanjing, Jiangsu, China). Test samples are dissolved in dimethyl sulfoxide (DMSO) (Sigma, USA) to get 100 mM, as a stock solution, and then stored at 4 °C. The final dilutions of DMSO added to the culture medium never exceeded 0.1% what insured there is no effect on cell viability.

### Cell culture

BV2 mouse microglia cells are originally developed by Chinese Academy of Sciences Shanghai cell bank and cultured in 25 or 75 cm^2^ flasks with Dulbecco’s modified Eagle’s medium (DMEM/25mM HEPES) (Gibco BRL, USA) supplemented with 10% fetal bovine serum (FBS) (Gibco BRL, USA), penicillin G (100 units/mL) and streptomycin (100 mg/mL) in a humidified incubator with 5% CO_2_/95% O_2_ at 37 °C.

### Cell viability assay

BV2 microglia cells are seeded into 96-well plate at a density of 1 × 10^5^ cells/ml, after incubated 18 h, cells are treated with 100 μl of fresh medium with or without various indicated concentrations of test samples for an additional 24 h. CCK-8 assay (BestBio, Shanghai, China) is a convenient, reliable method to determine viability of the cells. To eliminate the background of test samples, we discard the whole culture medium, after which 100 μl/well fresh media containing 10% CCK-8 solution is added then. The OD values at 450 nm are read on a microplate reader (Molecular Devices, California, USA) after a 3 h incubation at 37 °C and 5% CO_2_.

### Western blot analysis

The cellular protein is extracted from cell lines using a Qproteome™ Mammalian Protein Prep Kit (Qiagen, Germany) after the indicated procedures in accordance with the manufacturer’s protocol. Quick Stari Bradford Protein Assay Kit (Bio-Rad, USA) is applied to protein quantification. Equivalent amounts of protein (50μg) is denatured by boiling at 100 °C for 10 min with 2*laemmli sample loading buffer (Bio-Rad, USA) plus 5% β-mercaptoethanol in a ratio of 1:1 and loaded per lane onto 12% SDS-PAGE (sodium dodecyl sulfate polyacrylamide minigels), electrotransferred onto 0.45 μm polyvinylidene fluoride membranes (PDVF) (Millipore, Bedford, MA, USA) for 150 min at 200 mA. Subsequently, the membranes are blocked in 3% bovine serum albumin (BSA) at room temperature and incubated with the primary antibodies iNOS and COX-2 (Abcam) at 4 °C overnight. Following three thorough washes in Tris-buffered Saline-Tween (TBST) each for 5 min, the membranes are probed with horseradish peroxidase (HRP)-conjugated secondary antibodies (1:10000 dilutions; Abcam) for 1.5 h at room temperature. The immunoreactive bands are then visualized by using ECL chemiluminescence detection kit (Bio-Rad Laboratories, Richmond, California, USA) after washing twice in TBST and once TBS, each time for 5 min. Densitometric values are normalized using β-actin as loading internal control.

### Statistical analysis

Data are presented as means ± standard error, Western blot analysis are repeated three independent experiments with the same result. One-way analysis of variance is used to compare the differences of means for three or more groups, statistical significance is analyzed with the Student’s *t*-test between two groups.

### Equipment and settings

Excel of Microsoft Office2013 was used in Fig. 1.

Cytoscape 2.8.1 was used in the Fig. 2.

Cytoscape 2.8.1 was used in the Fig. 3.

Visio of Microsoft Office2013 was used in the Fig. 4.

Graphpad Prism 6 and Visio of Microsoft Office2013 were used in the Fig. 5.

### Data Availability

The datasets generated during and/or analysed during the current study are available from the corresponding author on reasonable request.

## Electronic supplementary material

Supplementary Tables

Supplementary Figures
