# Supplementary material for: In silico-based screen synergistic drug combinations from herb medicines: a case using Cistanche tubulosa
Source: Sci Rep. 2017 Nov 27;7:16364. doi: 10.1038/s41598-017-16571-3 (PMC5703970; doi:10.1038/s41598-017-16571-3)
Supplement: Supplementary file 1 — Supplementary Tables [file 41598_2017_16571_MOESM1_ESM.pdf]

In silico-based screen synergistic drug combinations from herb medicines: a case using *Cistanche tubulosa*

Jianling Liu[1†], Jinglin Zhu[1†], Jun Xue[1], Zonghui Qin[1], Fengxia Shen[1], Jingjing Liu[1], Xuetong Chen[2], Xiaogang Li[1], Ziyin Wu[2], Wei Xiao[3], Chunli Zheng[1*], and Yonghua Wang[1*]

Table S1. The information of the 103 compounds.

NO.	Name	DL
1	(2E,6Z)-8-β-D-glucopyranosyloxy-2,6-dimethyl-2,6-octadienoic	0.27
2	(2E,6Z)-8-β-D-glucopyranosyloxy-2,6-dimethyl-2,6-octadienoic_qt	0.04
3	2' -acetylacteoside	0.56
4	2' -acetylacteoside_qt_1	0.05
5	2' -acetylacteoside_qt_2	0.03
6	6-deoxycatalpol	0.39
7	6-deoxycatalpol_qt	0.09
8	8-epideoxyloganic acid	0.36
9	8-epideoxyloganic acid_qt	0.07
10	8-epiloganic acid	0.40
11	8-epiloganic acid_qt	0.09
12	8-epiloganin	0.44
13	8-epiloganin_qt	0.10
14	8-hydroxy geraniol 1-β-D-glucopyranoside	0.22
15	8-hydroxy geraniol 1-β-D-glucopyranoside_qt	0.03
16	8-hydroxy geraniol	0.03
17	20-hydroxyecdysone	0.83
18	Verbascoside	0.63
19	Antirrhide	0.29
20	Antirrhide_qt	0.05
21	Argyol	0.05
22	Bartsioside	0.29
23	Bartsioside_qt	0.05
24	Campneoside I	0.58
25	Campneoside I_qt	0.05
26	Cistachlorin	0.08
27	Cistanin	0.08
28	Cistanoside F	0.69
29	Cistansinenside A	0.54
30	Cistansinenside A_qt	0.04
31	Cistansinensose A1	0.65
32	Cistantubulose A1	0.61
33	Cistantubuloside A	0.40
34	Cistantubuloside A_qt	0.02

NO.	Name	DL
35	Cistantubuloside B1	0.40
36	Cistantubuloside B1_qt	0.04
37	Cistantubuloside C1	0.36
38	Cistantubuloside C1_qt	0.04
39	Decaffeoylacteoside	0.63
40	Dehydrodiconiferyl alcohlol -γ - O - β -D-glucopyranoside	0.85
41	Echinacoside	0.38
42	Geniposide	0.44
43	Geniposide_qt	0.10
44	Geniposidic acid	0.41
45	Geniposidic acid_qt	0.09
46	Gluroside	0.29
47	Gluroside_qt	0.05
48	Isoacteoside	0.60
49	Isosyringalide3'-α-L-rhamnopyranoside	0.66
50	Kankanol	0.09
51	Kankanoside A	0.32
52	Kankanoside A_qt	0.06
53	Kankanoside B	0.37
54	Kankanoside B_qt	0.08
55	Kankanoside C	0.39
56	Kankanoside C_qt	0.09
57	Kankanoside D	0.21
58	Kankanoside D_qt	0.03
59	Kankanoside E	0.26
60	Kankanoside E_qt	0.04
61	Kankanoside F	0.66
62	Kankanoside G	0.64
63	Kankanoside H1	0.36
64	Kankanoside I	0.44
65	Kankanoside I_qt	0.02
66	Kankanoside J1	0.52
67	Kankanoside K1	0.35
68	Kankanoside L	0.39
69	Kankanoside L_qt	0.09
70	Kankanoside M	0.30
71	Kankanoside M_qt	0.05
72	Kankanoside N	0.33
73	Kankanoside N_qt	0.06
74	Kankanoside O	0.27
75	Kankanoside O_qt	0.04
76	Kankanoside P	0.26
77	Kankanoside P_qt	0.04

NO.	Name	DL
78	Liriodendrin	0.29
79	Liriodendrin_qt	0.72
80	Mussaenosidic acid	0.40
81	Mussaenosidic acid_qt	0.09
82	Poliumoside	0.40
83	Salidroside	0.20
84	Syringalide A 3'-α-L-rhamnopyranoside	0.66
85	Tubuloside A	0.34
86	Tubuloside B	0.54
87	Tubuloside B_qt	0.04
88	Tubuloside C	0.23
89	Tubuloside D	0.24
90	Tubuloside E	0.60
91	β-sitosterol	0.75
92	(+)-syringaresinol O-β-D-glucopyranoside	0.77
93	(+)-syringaresinol O-β-D-glucopyranoside_qt	0.72
94	(3*R*)-3-hydroxy-1-methyl-2-pyrrolidinone	0.02
95	(3*R*)-3-hydroxy-2-pyrrolidinone	0.01
96	Ajugol	0.33
97	Ajugol_qt	0.07
98	Daucosterol	0.63
99	Daucosterol_qt	0.75
100	Jionoside D	0.61
101	Jionoside D_qt	0.70
102	Syringin	0.32
103	Syringin_qt	0.07

Table S2. Chemical information of 63 potential compounds and their network parameters.

Mol ID	Name	Structure	DL	Degree	Betweenness
mol01	20-Hydroxyecdysone	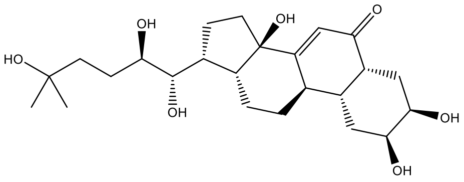	0.83	6	0.0118
mol02	Antirrhide	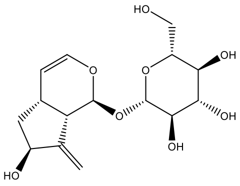	0.29	4	0.0008

Mol ID	Name	Structure	DL	Degree	Betweenness
mol03	Bartsioside	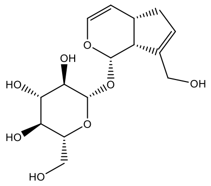	0.29	5	0.0019
mol04	Cistanoside F	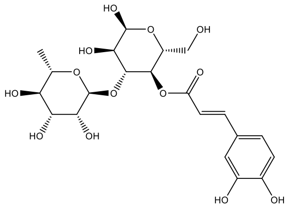	0.69	6	0.0086
mol05	(2E,6Z)-8-β-D-glucopyranosyloxy-2,6-dimethyl-2,6-octadienoic	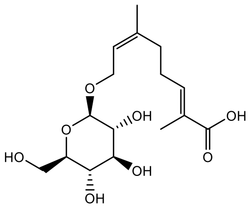	0.27	9	0.0098
mol06	Decaffeoylacteoside	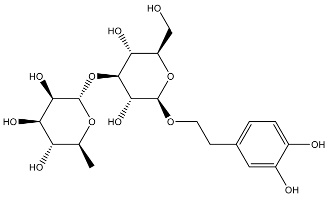	0.63	5	0.0053
mol07	Dehydrodiconiferyl alcohlol -γ - O - β -D-glucopyranoside	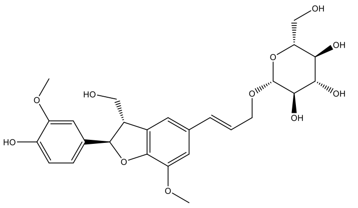	0.85	8	0.0173
mol08	Geniposide	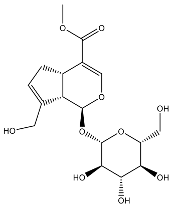	0.44	7	0.0052
mol09	Geniposidic acid	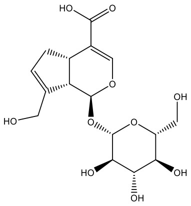	0.41	4	0.0021
mol10	Gluroside	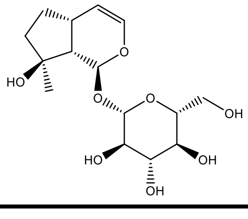	0.29	4	0.0008

Mol ID	Name	Structure	DL	Degree	Betweenness
mol11	Kankanoside A	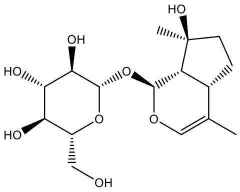	0.32	8	0.0115
mol12	Kankanoside B	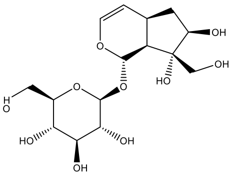	0.37	4	0.0008
mol13	Kankanoside D	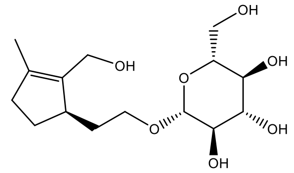	0.21	10	0.0164
mol14	Kankanoside E	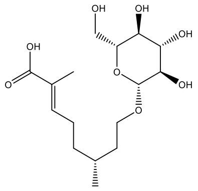	0.26	10	0.0129
mol15	Kankanoside L	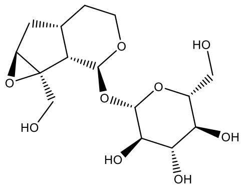	0.39	4	0.0039
mol16	Kankanoside M	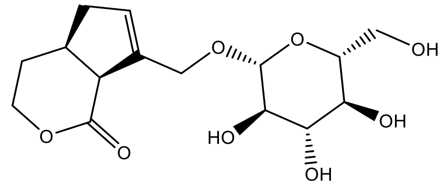	0.30	5	0.0019
mol17	Kankanoside N	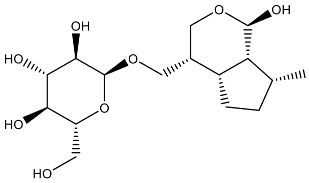	0.33	7	0.0047
mol18	Kankanoside O	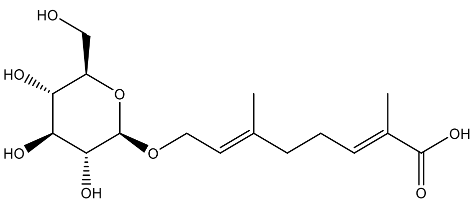	0.27	13	0.0284

Mol ID	Name	Structure	DL	Degree	Betweenness
mol19	8-Epideoxyloganic acid	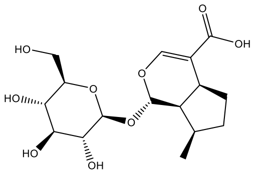	0.36	4	0.0008
mol20	Kankanoside P	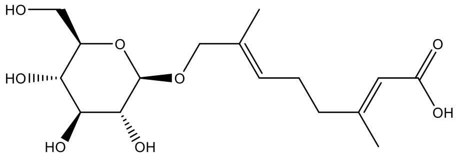	0.26	7	0.0052
mol21	Liriodendrin_qt	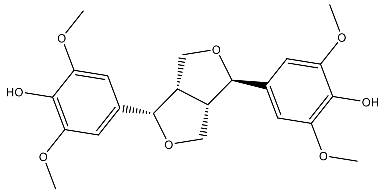	0.72	9	0.0147
mol22	Mussaenosidic acid	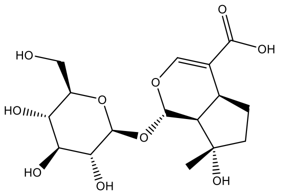	0.40	6	0.0043
mol23	Salidroside	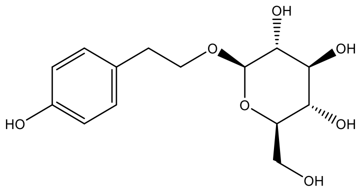	0.20	8	0.0324
mol24	8-Epiloganic acid	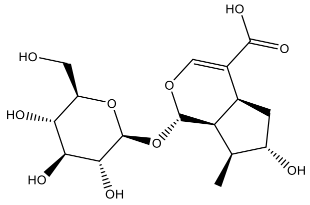	0.40	5	0.0028
mol25	β-sitosterol	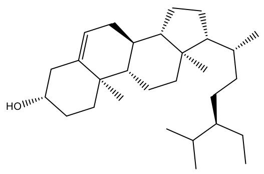	0.75	9	0.0188
mol26	(+)-syringaresinol O-β-D-glucopyranoside_qt	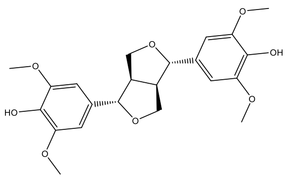	0.72	11	0.0375

Mol ID	Name	Structure	DL	Degree	Betweenness
mol27	Ajugol	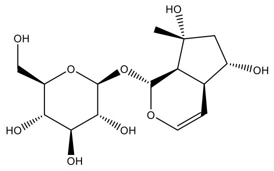	0.33	4	0.0008
mol28	Daucosterol_qt	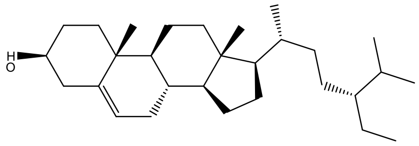	0.75	9	0.0188
mol29	Jionoside D_qt	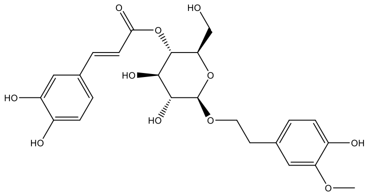	0.70	11	0.0423
mol30	Syringin	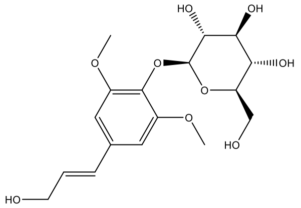	0.32	13	0.0704
mol31	8-Epiloganin	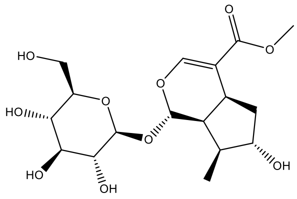	0.44	6	0.0038
mol32	8-Hydroxy geraniol 1-β-D-glucopyranoside	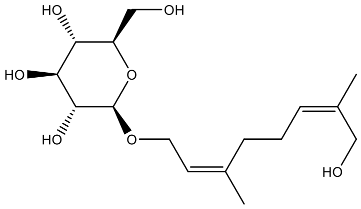	0.22	10	0.0168
mol33	Verbascoside	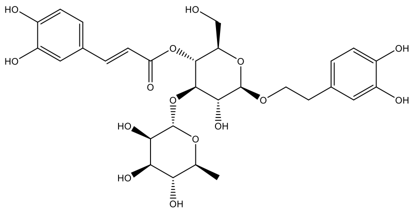	0.63	9	0.0110
mol34	Campneoside I	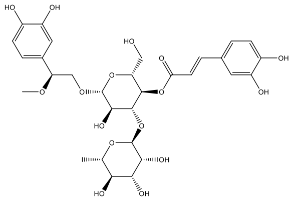	0.58	10	0.0136

Mol ID	Name	Structure	DL	Degree	Betweenness
mol35	Cistansinenside A	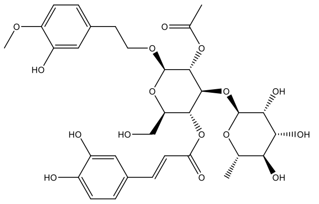	0.54	10	0.0136
mol36	Cistansinensose A1	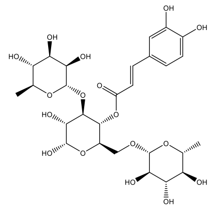	0.65	6	0.0068
mol37	Cistantubulose A1	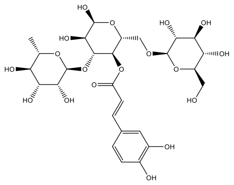	0.61	6	0.0068
mol38	Cistantubuloside A	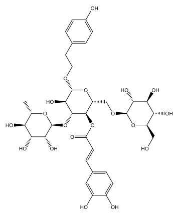	0.40	9	0.0063
mol39	Cistantubuloside B1	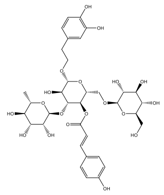	0.40	9	0.0063
mol40	Cistantubuloside C1	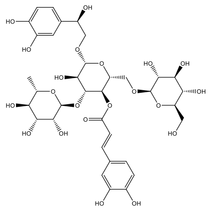	0.36	8	0.0125
mol41	Echinacoside	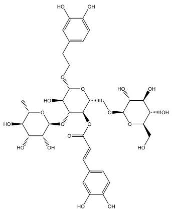	0.38	7	0.0016
mol42	2' -Acetylacteoside	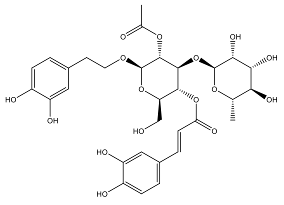	0.56	10	0.0136

Mol ID	Name	Structure	DL	Degree	Betweenness
mol43	Isoacteoside	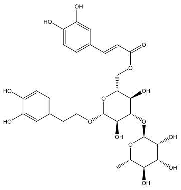	0.60	8	0.0420
mol44	Isosyringalide3'-α-L-rhamnopyranoside	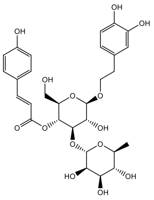	0.66	9	0.0110
mol45	Kankanoside C	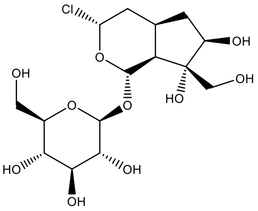	0.39	3	0.0026
mol46	6-Deoxycatalpol	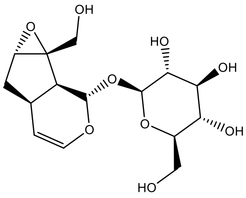	0.39	3	0.0026
mol47	Kankanoside F	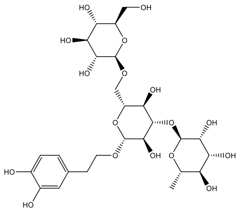	0.66	4	0.0003
mol48	Kankanoside G	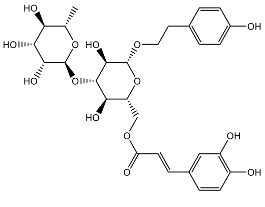	0.64	7	0.0053
mol49	Kankanoside H1	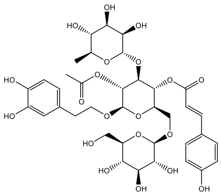	0.36	8	0.0021
mol50	Kankanoside I	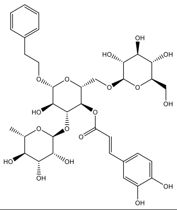	0.44	9	0.0045

Mol ID	Name	Structure	DL	Degree	Betweenness
mol51	Kankanoside J1	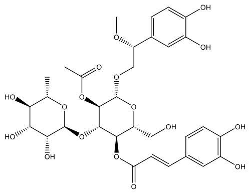	0.52	10	0.0136
mol52	Kankanoside K1	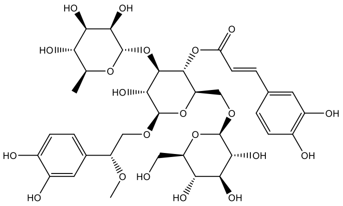	0.35	10	0.0199
mol53	Liriodendrin	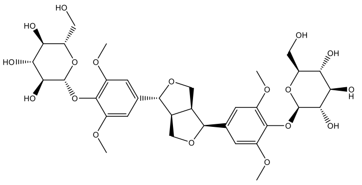	0.29	8	0.0304
mol54	Poliumoside	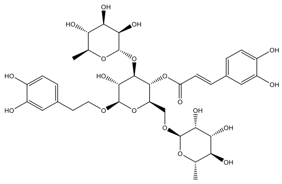	0.40	8	0.0021
mol55	Syringalide A 3'-α-L-rhamnopyranoside	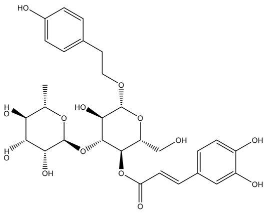	0.66	9	0.0110
mol56	Tubuloside A	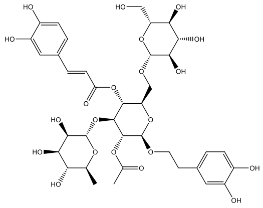	0.34	9	0.0063
mol57	Tubuloside B	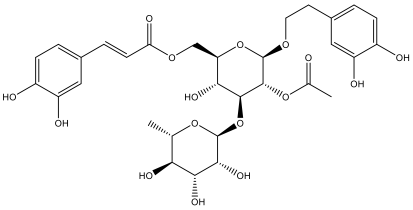	0.54	8	0.0065
mol58	Tubuloside C	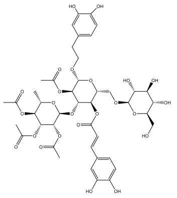	0.23	8	0.0100

Mol ID	Name	Structure	DL	Degree	Betweenness
mol59	Tubuloside D	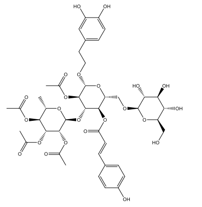	0.24	8	0.0100
mol60	Tubuloside E	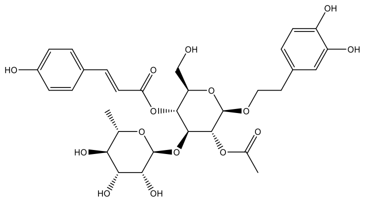	0.60	10	0.0136
mol61	(+)-syringaresinol-O-β-D-glucopyranoside	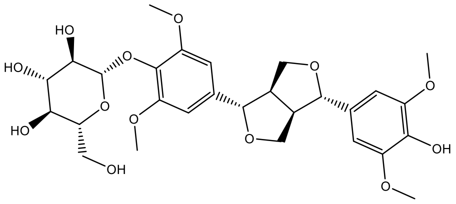	0.77	8	0.0133
mol62	Daucosterol	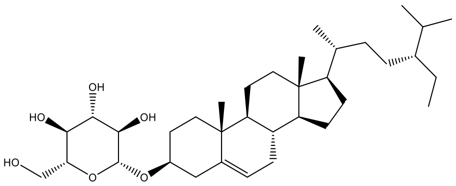	0.63	9	0.0681
mol63	Jionoside D	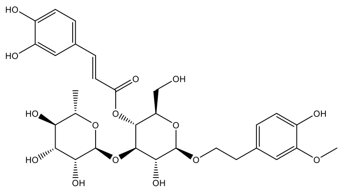	0.61	11	0.0186

Table S3. The information of the targets.

UniPort ID	Protein names	Gene	Degree
P16278	Beta-galactosidase	GLB1	47
P07686	Beta-hexosaminidase subunit beta	HEXB	37
P54577	Tyrosine--tRNA ligase, cytoplasmic	YARS	31
P08514	Integrin alpha-IIb	ITGA2B	30
Q9Y6L6	Solute carrier organic anion transporter family member 1B1	SLCO1B1	28
Q13451	Peptidyl-prolyl cis-trans isomerase FKBP5	FKBP5	28
P00918	Carbonic anhydrase 2	CA2	27
Q9UIQ6	Leucyl-cystinyl aminopeptidase	LNPEP	26
P04792	Heat shock protein beta-1	HSPB1	22
P35354	Prostaglandin G/H synthase 2	PTGS2	20
P03372	Estrogen receptor	ESR1	18
P00734	Prothrombin	F2	17
P28223	5-hydroxytryptamine receptor 2A	HTR2A	16

UniPort ID	Protein names	Gene	Degree
Q05655	Protein kinase C delta type	PRKCD	14
P10275	Androgen receptor	AR	12
P35228	Nitric oxide synthase, inducible	NOS2	11
P30989	Neurotensin receptor type 1	NTSR1	11
P37231	Peroxisome proliferator-activated receptor gamma	PPARG	9
Q08828	Adenylate cyclase type 1	ADCY1	7
P42261	Glutamate receptor 1	GRIA1	7
P10636	Microtubule-associated protein tau	MAPT	6
P05067	Amyloid beta A4 protein	APP	6
P29474	Nitric oxide synthase, endothelial	NOS3	5
P11473	Vitamin D3 receptor	VDR	5
P50052	Type-2 angiotensin II receptor	AGTR2	5
O15528	25-hydroxyvitamin D-1 alpha hydroxylase, mitochondrial	CYP27B1	4
P22303	Acetylcholinesterase	ACHE	3
P30536	Translocator protein	TSPO	3
Q99835	Smoothened homolog	SMO	3
Q9BVA1	Tubulin beta-2B chain	TUBB2B	3
P05093	Steroid 17-alpha-hydroxylase/17,20 lyase	CYP17A1	3
P19793	Retinoic acid receptor RXR-alpha	RXRA	3
Q12809	Potassium voltage-gated channel subfamily H member 2	KCNH2	2
P62158	Calmodulin	CALM1	2
P06401	Progesterone receptor	PGR	2
P06881	Calcitonin gene-related peptide 1	CALCA	2
P20248	Cyclin-A2	CCNA2	1
P08253	72 kDa type IV collagenase	MMP2	1
P14780	Matrix metalloproteinase-9	MMP9	1
Q15661	Tryptase alpha/beta-1	TPSAB1	1
Q13224	Glutamate receptor ionotropic, NMDA 2B	GRIN2B	1
P16109	P-selectin	SELP	1
P23219	Prostaglandin G/H synthase 1	PTGS1	1
