# Supplementary material for: In silico-based screen synergistic drug combinations from herb medicines: a case using Cistanche tubulosa
Source: Sci Rep. 2017 Nov 27;7:16364. doi: 10.1038/s41598-017-16571-3 (PMC5703970; doi:10.1038/s41598-017-16571-3)
Supplement: Supplementary file 2 — Supplementary Figures [file 41598_2017_16571_MOESM2_ESM.pdf]

In silico-based screen synergistic drug combinations from herb medicines: a case using *Cistanche tubulosa*

Jianling Liu[1†], Jinglin Zhu[1†], Jun Xue[1], Zonghui Qin[1], Fengxia Shen[1], Jingjing Liu[1], Xuetong Chen[2], Xiaogang Li[1], Ziyin Wu[2], Wei Xiao[3], Chunli Zheng[1*], and Yonghua Wang[1*]

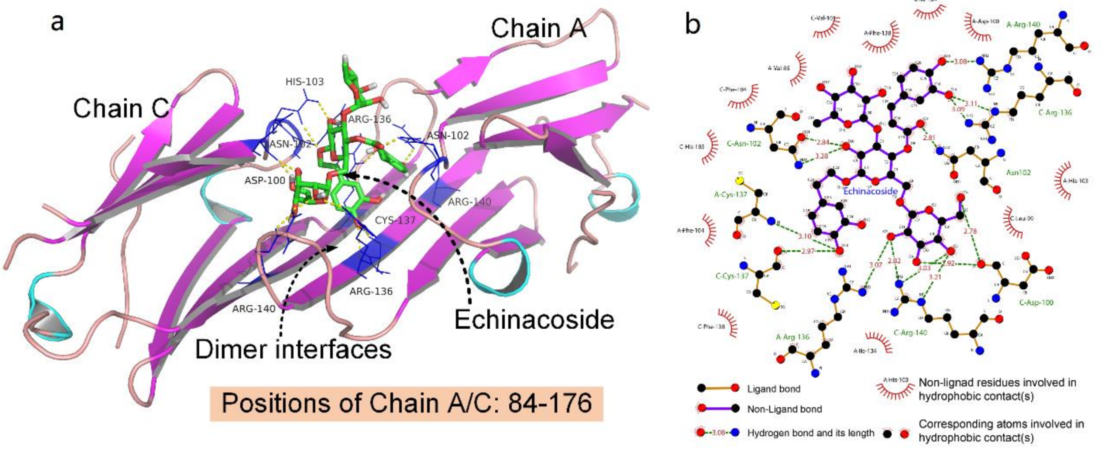

Supplementary Figure 1. Molecular models of the echinacoside in the binding sites of HSPB1. (a) 3D ligand-protein interaction diagram between echinacoside and HSPB1. The dashed lines (yellow) show the formation of the hydrogen bonds. Active site amino acid residues are represented as lines (blue). (b) 2D ligand-protein interaction diagrams between echinacoside and HSPB1. The dashed lines (green) show the formation of the hydrogen bonds.

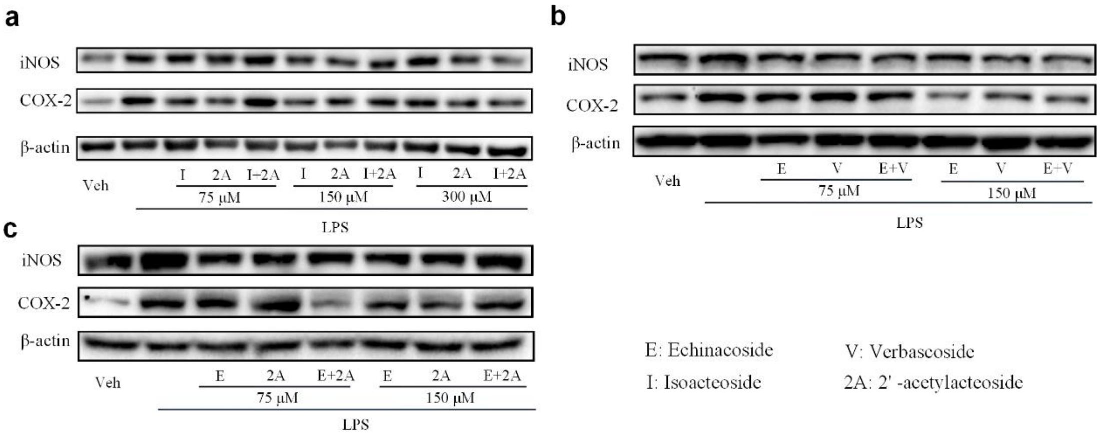

Supplementary Figure 2. Inhibition of iNOS and COX-2 in BV2 cells. (a-c) BV2 cells are pretreated with (a) I or 2A (75, 150 or 300 μM), (b) E or V (75 or 150 μM), and (c) E or 2A (75 or 150μM) or the combinations for 2 h, vehical as the control. Then exposure to LPS (1 μg/ml) for 18 h, then iNOS and COX-2 accumulation of cytoplasm are measured by western blot. β-actin is used as loading control.
